# Learning end-of-life care within a constructivist model: Undergraduate nursing students**’** experiences

**DOI:** 10.4102/curationis.v38i2.1537

**Published:** 2015-11-05

**Authors:** Anna E. van der Wath, Pieter H. du Toit

**Affiliations:** 1 Department of Nursing Science, University of Pretoria, South Africa; 2 Department of Humanities Education, University of Pretoria, South Africa

## Abstract

**Background:**

Although nursing education aims to equip nursing students to provide care to dying patients and their families, nurses often feel ill-prepared to cope with the emotional labour involved in end-of-life care.

**Objectives:**

The aim of the study was to explore and describe nursing students’ experiences of end-of-life care through experiential learning within a constructivist educational model.

**Method:**

A qualitative, descriptive design was used. As part of introducing experiential learning, innovative educational practices were initiated during a second year level undergraduate nursing module on end-of-life care. Qualitative data on second-year nursing students’ experiences were collected through written reflections and analysed using open coding.

**Results:**

The themes that emerged revealed participants’ sensory and emotional experiences during the learning opportunities. Participants reflected on what they learnt and clarified their values related to death and dying. They indicated how they would apply the new meanings constructed in clinical practice.

**Conclusion:**

A constructivist educational model of experiential learning holds potential to enhance value clarification and nursing students’ sensory and emotional awareness of death and dying. Experiential learning is recommended to develop nursing students’ competency in providing end-of-life care.

## Introduction

Learning how to face death and dying is inevitable for nurses who are often the caregivers at the end-of-life (Todaro-Franceschi & Lobelo 2014:171). Mastering end-of-life care competence often confronts nursing students with their own mortality and their personal beliefs about death and dying (Peters *et al.* 2013:14).

To facilitate learning of end-of-life-care is also a challenging task for nurse educators (Dobbins 2011:159). Death and dying is a sensitive topic and educators may feel ‘cautious and over-conscious’ regarding the emotional side of end-of-life care (Venkatasalu, Jackson & Walsh 2014:A34). Chan and Tin (2012:900) believe that to prepare nurses for the demands of end-of-life care requires more than the traditional teaching methods that focus on transferring knowledge and skills. They believe that ‘self-competence’, referring to personal resources, existential coping, and emotional coping, is a necessary competency to deal with death, dying and bereavement (Chan & Tin 2012:899). Experiential learning, which includes self-reflection to integrate and apply knowledge, skills and self-competence, is recommended as s strategy to facilitate self-competence in end-of-life care (Chan & Tin 2012:909–910). Value clarification, a process of self-reflection to identify and consider personal beliefs about death and the worth of life, may also help to prepare nurses for ethical decision-making during end-of-life care (Tarzian & Schwarz 2010:35–36).

## Background

There is a clear call for a paradigm shift in nursing education. Instead of lecture-based teaching where students are passive recipients, models and methods enhancing active student participation are proposed (Brandon & All 2010:89; Freed & McLaughlin 2013:174; Rieger & Chernomas 2013:53; Stanley & Dougherty 2010:378). Brandon and All (2010:89) proposed a constructivist paradigm to enhance nursing student-centred interactive learning. An example of this is a reflection-centred curriculum designed to enhance the intra- and interpersonal development of nursing students (Horton-Deutsch, McNelis & O’ Haver Day 2012:341).

Nursing care requires nurses to be emotionally competent and able to deal with others’ emotions, whilst regulating their own (Vilelas & Diogo 2014:147–148). In end-of-life care, nursing education should make provision for nursing students to experience and manage their own emotional reactions towards death and dying. Gillan, Van der Riet and Jeong (2014:341) recommended that educators consider students’ personal and professional experiences with death and dying and to provide different learning opportunities to meet all students’ needs during end-of-life care education.

### Literature review

In a study by Todaro-Franceschi (2011:318) professional nurses indicated that their training did not equip them with the competence to care for the dying and their families (Todaro-Franceschi 2011:318). Twenty five per cent of oncology nurses felt they were inadequately prepared to care for dying patients (White & Coyne 2011:714). Professional nurses in New Zealand reported that their early experiences with death and dying, for which they felt unprepared, left a lasting personal and professional impression. They experienced helplessness, distress and guilt when a patient died (Kent, Anderson & Owens 2012:1255). Fear of death and a negative attitude towards end-of-life care is more evident in younger nurses (Peters *et al.* 2013:14). When feeling unprepared to deal with death and dying, nursing students may avoid emotional involvement with dying patients (Mutto *et al.* 2012:97). Nurses who experience challenges in caring for dying patients may experience poor professional quality of life and increased risk for compassion fatigue and burnout (Todaro-Franceschi & Lobelo 2014:170).

A literature review by Gillan *et al.* (2014:341) found that nursing students feel unprepared to deal with death and dying and schools of nursing are not adequately preparing nursing students for end-of-life care, leading to inadequate care once they are employed. The findings highlighted the lack of end-of-life content in textbooks and lack of content on end-of-life care in undergraduate nursing curricula. Venkatasalu *et al.* (2014:A34) described this phenomenon as ‘hidden death’ which relates to the ‘lack of explicit curricular objectives around end-of-life care topics’ that ‘results in limited exposure to death and dying topics in pre-registration programmes’. They also point out a lack of support structures to help educators and students deal with the emotional issues surrounding end-of-life education (Venkatasalu *et al.* 2014:A34).

Many examples of experiential opportunities to facilitate learning for end-of-life care are available in scholarly literature. Dobbins (2011:161) mentioned ‘cinemeducation’, the use of movies or movie clips, as a method to incorporate an educational experience that serves as an opportunity for critical self-reflection. Self-awareness and self-reflection during facilitating learning are also reported to have helped students to gain experiential knowledge to provide end-of-life care (Liu *et al.* 2011:861). Real-life cases and open-group discussions have been reported to facilitate emotional intelligence, the management of emotional labour and exploration of personal emotions (Bailey & Hewison 2014:3555). Storyboarding, a process that engages the creative right brain and the critical left brain, has helped students to identify cultural aspects and feelings related to the dying patient (Lillyman, Gutteridge & Berridge 2011:179). Two experiential educational strategies, high fidelity and gaming simulation, were also used by Kopp and Hanson (2012:e99–e101) in end-of-life care education to help students develop awareness of the issues surrounding terminal illness and the importance of having empathy with these patients. In this study, students verbalised increased awareness of the losses patients suffer, whilst considering how they would respond in a similar situation. Another example is how a workshop enhancing students’ reflections on life and death helped them to gain experiential knowledge on end-of-life care, cherish life and explore their feelings related to facing death (Liu *et al.* 2011:860).

Experiential learning is described as a philosophy and a teaching method that focuses on the relationship between students, their concrete experiences and their reflective processes about the experience (Fenwick 2001:16). As described by Kolb 1984:

To learn is not the special province of a single specialized realm of human functioning such as cognition or perception. It involves the integrated functioning of the total organism-thinking, feeling, perceiving and behaving. (p. 31)

Experiential learning stems from Schön’s ‘epistemology of practice’ (1983:49, 66–68), Mezirow’s transformative learning theory (Mezirow 1991:7–8, 14, 77), the phenomenological perspectives of Boud, Cohen and Walker (1993:8–16) and Kolb’s cycle of experiential learning (1984:33).

The constructivist view underpins experiential learning (Fenwick 2001:9). Constructivism assumes that learning takes place as students create new meanings which are integrated with existing constructs (Kanselaar 2002:1). Stemming from the theories of Vygotsky (1896–1934), socio-constructivists add to constructivist assumptions by viewing learning as a social process that occur through engagement in social interactions (Vygotsky 1978:38). In a constructivist view of experiential learning, students are believed to interpret their worlds actively and construct knowledge through processes of reflection (Fenwick 2001:10, 14). A constructivist model of experiential learning was developed by Kolb (1984:33). The student learns through cycles of concrete experience (a simulated or real-life experience), reflective observation (questioning the observations, awareness and meaning of the experience), abstract conceptualisation (reflecting on principles and new understandings) and active experimentation (reflecting on future application by revising and reshaping the learning) (Fenwick 2001:10–11; Kolb 1984:30–33).

### Problem statement

End-of-life care is considered to be a complex area of practice in intensive care units, and nurses expressed the need for emotional support (Ranse, Yates & Coyer 2012:14). Research indicated that undergraduate nursing students often feel unprepared to deal with dying patients and their families (Gillan *et al.* 2014:331).

Teaching and learning that focus on knowledge and skills development only is not considered the most effective way to equip nursing students to cope with the emotional and existential challenges of end-of-life care (Chan & Tin 2012:900). Insufficient focus on emotional aspects may result in students feeling ill-prepared to care for dying patients and their families (Bailey & Hewison 2014:3555–3560). When students are involved in purposefully designed educational strategies that focus on knowledge, skills and attitudes necessary to provide compassionate end-of-life care, they will be better prepared to deal with the bio-psychosocial and spiritual needs of dying patients (Dobbins 2011:165). Nurse educators need to find effective strategies of facilitating learning to prepare nursing students for care of dying patients (Dobbins 2011:159). Nursing education that focuses on self-awareness and own beliefs and emotions regarding death and dying seem to have a positive effect on students’ attitudes towards end-of-life care (Bailey & Hewison 2014:3555–3560).

Gillan *et al.* (2014:341) identified a lack of qualitative research on end-of-life teaching and the need to consider students’ experiences with death and dying during the facilitation of learning. Venkatasalu *et al.* (2014:A34) are of the opinion that further research to evaluate end-of-life care strategies of facilitating learning and how these strategies relate to clinical care outcomes, is needed.

## Aim of the study

The aim of the study was to explore and describe nursing students’ experiences regarding end-of-life care through experiential learning within a constructivist model.

## Definition of key concepts

*Constructivism* assumes that there are multiple representations of reality. Knowledge is constructed through collaborative participation in authentic tasks in real-life environments and reflection on these experiences (Jonassen 1999:217–219). In this article ‘constructivism’ refers to an experiential learning model where students are believed to construct knowledge through collaborative participation and processes of reflection.

*Experience* was described by Dewey, an influential educational theorist of the twentieth century, as a process of interaction ‘going on between an individual and objects and other persons. An experience is always what it is because of a transaction taking place between an individual and what, at the time, constitutes his environment …’ (Dewey 1938:43). In this article ‘experience’ refers to the interaction taking place in the environment where nursing students learn to provide end-of-life nursing care.

*End-of-life-care nursing education* aims to increase nursing students’ understanding of patient care for dying patients and the related ethical-legal, cultural and spiritual issues. It also cultivates a respectful attitude towards death (Liu *et al.* 2011:857). In this article ‘end-of-life care nursing education’ refers to a module in the second year where undergraduate nursing students learn to provide care to dying patients and their families.

*Undergraduate nursing students* in this article refer to second-year nursing students at a higher education institution in Gauteng Province enrolled for a degree in nursing and registered as learners at the South African Nursing Council (2005:27).

## Contribution to field

The study findings may contribute to nursing education, practice and research. The article illustrates the use of a constructivist model of experiential learning (Kolb 1984:33) in end-of-life care nursing education for undergraduate nursing students. The findings may help to understand the ways in which the learning experiences contribute to sensory, cognitive and emotional awareness; value clarification and nursing students’ perceived competencies regarding end-of-life care. The innovative educational practices introduced in the article may help to ensure nurses feel more prepared to provide end-of-life care to patients and their families. Finally, this research may contribute to the research-based body of knowledge on end-of-life nursing care.

## Research design and methods

### Design

A qualitative design (Polit & Beck 2012:14) was used to explore and describe nursing students’ experiences of end-of-life care through experiential learning within a constructivist educational model.

A psychiatric nursing module for second-year students, dealing with end-of-life care, was used to integrate innovative educational practices such as co-operative learning and reflective processes. Opportunities were created to engage students in experiential learning. Students were divided into co-operative learning groups. Each group was assigned the learning task of presenting their experiences on a theme related to death and dying based on an applicable film, an article, a story, a poem or a case study of their choice. Students used a variety of methods (demonstrations, acts, posters, video clips and mind maps) to present the themes during four learning opportunities as scheduled on the timetable. Each presentation was followed by an open class discussion where applicable theoretical models and practical interventions were introduced, for example, ‘breaking the bad news’.

### Participants

Sixty four students registered for the second year of a four-year undergraduate nursing science programme in a higher education institution constituted the population for the study. All the students participated in the experiential learning opportunities and were invited to write reflective narratives on the experience of learning of end-of-life care. Sixty two students voluntarily handed in written reflections.

### Data collection method

Qualitative data were collected through the use of students’ written reflections which served as a narrative account of their experiences (Mendieta 2013:140). To facilitate deep self-reflection, Schön’s model of reflection (1983:26, 61–62) was used as a guideline. The model explicates reflection-in-action as a way of attending to experiences and coming in touch with feelings to create new understanding. Reflection-on-action is done after the experience to explore and think about actions. Students were requested to write a reflection during each learning opportunity, using a layout based on the acronym, LEARN (College of Nurses of Ontario 1991, in Brown, Matthew-Maich & Royle 2001:133):

- Look back on the situation.
- Examine the detail: What happened? How were you involved? How did you feel? What were you thinking? Who else was involved? What did others say?
- Analyse: What influenced the experience? What role did the values of different people play? What have you learned? Will it influence your practice?
- Revise: What would you do differently next time? Are there any learning issues? How can you use what has been learned? What needs to change?
- New perspective: Recommendations for learning in similar future situations.

### Data analysis

Qualitative data analysis of written reflections was done according to the steps as described by Tesch (cited in Creswell 2009:85). The researcher read through the data to obtain a general sense of the total experience and reflected on the meanings students attached to the learning opportunities. Text sections were segmented and themes labelled with appropriate terms. The themes were described as derived from the data and interrelationships between the themes noted. Guided by the principle of data saturation, written reflections were analysed until no new themes emerged (Polit & Beck 2012:742).

### Context of the study

The research setting constitutes a four-year undergraduate nursing science programme in a higher education institution in Gauteng Province, South Africa.

This programme includes a module on end-of-life care in the second year.

## Results

The themes ‘I saw’, ‘I heard’ and ‘I did’ that emerged from participants’ reflective narratives illustrate their sensory experiences during the learning opportunities. The feelings participants experienced during the presentations are reflected in the theme, ‘I felt’. The theme ‘I learnt’ indicate what and how participants learnt. The theme ‘I clarified my values’ reveals participants’ reflections on their values related to death and dying. Participants applied what they have learnt to practice: what ‘I will do’ differently next time when encountering death and dying in clinical practice. The results are discussed according to these themes, supported by excerpts from different students’ verbatim narratives.

### ‘I saw’

Different participants specifically mentioned that the visual material enhanced their learning as in the following examples:

‘We looked at various videos and we watched various physical performances by our classmates. In these, various principles of how to deal with grief and how grief present itself, was presented.’ (Participant 15, male)

‘The experience was influenced by the use of video clips, visual learning made it easy for me to relate the topic with the actual reality.’ (Participant 22, female)

‘I found the videos are an effective way of showing us how children grieve in a visual way – a way that can easily be remembered.’ (Participant 30, female)

‘Different groups drew pictures and made posters to help us visualise the process.’ (Participant 46, female)

Participants who observed other students demonstrating some of the skills indicated that it also enhanced learning:

‘I think the acting out part was also helpful because most of the time what you have seen is not easily forgotten.’ (Participant 12, female)

‘Making the students demonstrate the management allows other learners to learn in a more realistic way.’ (Participant 35, male)

Participants observed each other’s emotional expressions during the learning opportunities and sensed the emotional atmosphere elicited by the presentations:

‘… expression of sadness could be seen through their faces, especially the group members.’ (Participant 5, male)

‘Students that were sitting next to me were smiling because of the wonderful lessons that we got.’ (Participant 32, female)

‘The rest of the class also became quiet and depressed.’ (Participant 43, female)

### ‘I heard’

Some participants (fewer than in the ’I saw’ group) mentioned that hearing – hearing music, poetry and others’ experiences – enhanced their learning experience:

‘The issue of calm music is also recommended to facilitate the audience’s emotions.’ (Participant 12, female)

‘I appreciated the music to explain the class content. I feel music is a great indirect way of explaining a subject.’ (Participant 24, female)

‘In poems and scenarios read we witnessed different maladaptive behaviours that people manifest.’ (Participant 41, female)

‘I was listening.’ (Participant 52, female)

‘Others express a feeling of sadness, by shouting out or verbalising words like hah.’ (Participant 60, female)

### ‘I did’

Participants commented on and appreciated their own and others’ participation and involvement in the co-operative learning opportunities:

‘We spoke about principles in management of grief … about different ways in which children may respond … different cultural needs … I was involved in the presentation.’ (Participant 8, female)

‘I was involved by giving the feedback on each topic discussed; this made me feel more involved.’ (Participant 13, female)

‘Class interaction is very crucial.’ (Participant 28, female)

‘I read out the case study and applied the learning content … I felt good because I at least contributed something in the class.’ (Participant 36, female)

‘My other group members were involved.’ (Participant 42, female)

‘Others said they have also been in similar situations.’ (Participant 55, female)

‘We got to participate, do some presentations and acting. This helped me to concentrate throughout the lecture and to be willing to know more.’ (Participant 58, female)

‘At first I was shy until I got involved and gain my confidence.’ (Participant 60, female)

### ‘I felt’

Most participants mentioned their emotional experiences during the learning opportunity as significant. The learning material triggered intrapersonal experiences of loss and bereavement:

‘During the presentations I felt very interested in what was said, but some of the clips got me emotional. Because grief is a difficult subject to understand as people experience it so differently, I felt a level of sadness.’ (Participant 4, female)

‘… they really dragged my emotions into reality.’ (Participant 13, female)

‘Well during the presentation I got emotional … I have been faced with the loss of two grandparents … so I could relate somehow.’ (Participant 22, female)

‘A young girl lost her close friend in a movie, it made me feel so sad and I started imagining myself losing the one I love then I cried. Others said it’s good to cry.’ (Participant 28, female)

‘I was thinking how I reacted or it just reminded me of the loved one that I have lost few years back … my friends and I and the whole class was involved in the class discussion.’ (Participant 39, female)

‘I also related to the grief because I also experienced the same stages of grief.’ (Participant 53, female)

‘It brings back old memories of what happened to me … I almost cried during class discussion.’ (Participant 58, female)

Some participants felt guilty for getting emotionally involved:

‘And next time I will try not to identify myself with the clips we view in class.’ (Participant 10, female)

‘Next time I will make sure that I try to be as objective as possible and that I do not let my emotions overwhelm me.’ (Participant 24, female)

‘Next time I will listen effectively, I won’t let my past interfere with my attention … I want to learn about death without being sad.’ (Participant 36, female)

### ‘I learnt’

Participants expressed their appreciation for the way in which learning was facilitated:

‘The effective manner in which learning was enhanced leaves me with no idea of how I could change anything.’ (Participant 15, male)

‘I have experienced that if the lessons are taught with the use of presentations, some acting and some pictures it gives a lot of understanding and it is not easy to forget.’ (Participant 23, female)

‘I have learnt a world’s worth of information on reflection and feel that I have grown in leaps and bounds as a person. Thank you!’ (Participant 49, female)

Participants also experienced the joy of learning:

‘I enjoyed the acts the students put on because they made it funny and I think it will be easy for me to remember.’ (Participant 12, female)

‘Thank God for school! I really learnt a lot.’ (Participant 16, female)

‘Videos, case studies and presentations made the learning fun and understandable.’ (Participant 37, female)

‘The topic was very emotional and sensitive; however some plays made us laugh and move away from the sorrow.’ (Participant 50, female)

New meaning was constructed:

‘… as the class went on and I learnt more skills; I saw it is possible to be there for someone who is grieving.’ (Participant 11, female)

‘I now better understand why I need to go through the stages [*of grief*] to process all the emotions.’ (Participant 32, female)

‘I never understood why children act differently … what I’ve learned I’ll be able to use throughout my life.’ (Participant 37, female)

‘I never considered how much different these processes are for children than for adults.’ (Participant 54, female)

‘Death can have a calming or beautiful effect when done in a gracious and gentle manner.’ (Participant 56, female)

### ‘I clarified my values’

Participants shared the existential intelligence they obtained through the learning opportunities:

‘What I should do differently is to talk about death. I was afraid of talking about it previously. I have realised that if we are afraid of death we are afraid of life itself.’ (Participant 14, female)

‘Dying is part of life. We all get born and live our lives and eventually will die.’ (Participant 47, female)

They also obtained interpersonal intelligence on how to appreciate and respect others’ values with regards to death and dying:

‘I will be more understanding and empathic … I will have more respect for people and how they grief.’ (Participant 11, female)

‘I will not bring my own frame of reference into other people’s situations especially when they experience loss.’ (Participant 36, female)

‘The clip that was shown was very beautiful and showed the respect that was needed to take care of someone that died.’ (Participant 48, female)

### ‘I will do’

Most participants were able to relate the learning to their clinical practice and how it will affect the care they provide. They verbalised feeling more competent to deal with challenging clinical situations:

‘… we should actually give time to people that are grieving and be there for them … this will influence the way I view other people’s culture … I won’t judge.’ (Participant 2, female)

‘I learnt a lot about dying, how to break the bad news, what to say and not to say.’ (Participant 7, female)

‘I now, however feel I can provide the support I need to give to a grieving patient.’ (Participant 15, male)

‘I will comfort the grieving family members and not judge them.’ (Participant 22, female)

‘… principles that can help each of us to be caring and helpful.’ (Participant24, female)

‘Compassion is important.’ (Participant 31, female)

‘… it is important to positively regard other people’s feelings of loss as important.’ (Participant 47, female)

‘I can use the lessons to different culture groups, in the children’s wards because I now have a knowledge of the process of grieving.’ (Participant 48, female)

‘Don’t ever deny people the opportunity to cry and go through grief … don’t always feel the necessity to say something. If you have nothing to say just reflect.’ (Participant 55, female)

## Ethical considerations

Permission to conduct the research was obtained from the Research Ethics Committee (Information removed to ensure blind peer review). The principles of the Belmont Report (Polit & Beck 2012:152–253) assisted in avoiding exploitation of participants and ensured that their rights were protected.

### Potential benefits and hazards

Students benefited directly from the research, and the findings may contribute to educational nursing practice. Students were invited to discuss any emotional discomfort and personal experiences they experienced during participation in private with the researcher, a psychiatric nursing specialist, or with the student advisor who is available to all students at the faculty. Two students made use of the opportunity and shared the way they benefited from the learning opportunity as it facilitated their own grieving. They did not require referral for emotional support.

### Recruitment procedures

Students were invited and given freedom to choose to either participate in the study or discontinue at any given time without the risk of incurring adverse consequences. Students had an equal chance to be included in and benefit from the research.

### Informed consent

Participants were provided with a participant information leaflet to explain the nature of and likely consequences of the research and signed informed consent prior to participation.

### Data protection

Reflective reports were assigned a code number to ensure confidentiality. Reflective reports will be stored for 15 years in a safe place at the Nursing Science Department.

## Trustworthiness

Lincoln and Guba’s model (cited in Polit & Beck 2012:589) for assessing the trustworthiness of data was applied. Credibility was obtained through spending sufficient time with students to generate an in-depth understanding of their experiences of the learning material and opportunities. The researcher reflected on any personal values that could have affected data collection and analysis by keeping a reflective journal to stay in contact with her own experiences. To obtain transferability and dependability, sufficient descriptive information of the methodology and setting were provided to allow for comparison should readers be interested to apply the findings to similar situations. Confirmability was obtained through keeping the raw data as supportive evidence of the findings. Authenticity, the extent to which the research fairly and faithfully shows a range of realities, was obtained by supporting the findings with students’ narratives.

## Discussion

### Outline of the results

Video clips, music, demonstrations, posters, case studies, poems and pictures were used during the learning opportunities. The participants reflected on their sensory (visual, auditory and kinaesthetic) and emotional experiences. They experienced co-operative learning in groups (Johnson, Johnson & Stanne 2000:2) and reflected on their own learning. The quotes reflect the meaning participants constructed about death and dying. Some participants clarified the values they attached to death and dying. They reflected on how they would apply the new meaning they constructed in clinical practice, whilst some of them perceived themselves as more competent to deal with death and dying.

Participants in this research identified certain competencies they acquired during the learning opportunities. Chan and Tin (2012:899) identified similar competencies that health care professionals require when they are working with death, dying and bereavement. The competencies included knowledge (cognitive), practical (clinical) experience and self-competence which requires personal resources, emotional and existential (value-driven) coping. The same authors (2012:909) similarly used creative and interactive means such as movies, videos, songs and group discussions, to facilitate helping professionals’ preparation for what they called ‘death work’.

Sensory awareness enhances learning. The senses offer a means of contact and communication with the external world, but the raw sense data need to be constructed through perception (Beard & Wilson 2013:166–167). Whilst experiential learning is about observing, reflecting and doing, the three dominant thinking patterns – visual, auditory and kinaesthetic (Beard & Wilson 2013:177, 179) – are evident in students’ reflections on the learning opportunities they were exposed to. The concept of ‘embodied pedagogy’ is described by Nguyen and Larson (2015:n.p.) as learning that takes place through a ‘bodily and spatial awareness of sensation and movement’ and mind-body interaction in a ‘socio-cultural context’. The authors asked the question of how to engage the body, the reflective mind and the social being of the student in learning to construct knowledge and meaning. The findings in this article illustrate how participants used their bodily senses to engage in learning and construct new meaning.

Confrontation with death and dying, be it a direct or indirect experience, elicits emotions. The participants acknowledged their feelings of sadness during the experiential learning. The encounter with dying in clinical practice may trigger more than sadness. For example, medical and nursing students in a study in Argentina described feelings of helplessness, vulnerability and sympathy for patients (Mutto *et al.* 2012:95). Students given the opportunity to experience and reflect on their emotional responses in a safe and supportive environment begin to learn how to manage their emotions related to death and dying. Identification of emotional labour is the first step towards emotional intelligence which has a positive effect on nurses’ attitudes towards end-of-life care (Bailey & Hewison 2014:3561–3562). A literature review revealed that nurses’ unresolved personal experiences with death can play a role in the way they approach death and dying (Wilson & Kirshbaum 2011:559).

Nursing curricula that incorporated competencies associated with emotional intelligence positively related to students’ well-being and effective stress-management skills. Emotional intelligence focuses on self-awareness and identifying and monitoring own and others’ emotions to guide thoughts and behaviour (Por *et al.* 2011:856, 859, 860). Nurses’ empathetic capacity may also be enhanced through emotional intelligence (Williams & Stickley 2010:754).

Some students who participated in the study reported in this article felt guilty about getting emotionally involved; this might be a product of socialisation or the expectation that nurses must suppress their emotions. In the class discussion the students had the opportunity to talk about emotional expression: If to be encouraged in patients, why do nurses have to feel guilty when they experience sadness and pain? A study by Jack and Wibberley (2014:905–906) describe the complex emotional needs of nursing students who felt the need to change on an emotional level, but struggled to find ways to deal with the emotional demands of nursing. Although they wished to engage in discourse to find meaning in the emotional experiences they encountered, they felt lost and unable to share their feelings. The authors emphasise the need to find ways to facilitate student nurses’ emotional coping.

Participants in the study also experienced positive emotions as they expressed their appreciation for the learning opportunities. A storyboarding technique to address end-of-life experiences in practice yielded positive experiences as students appreciated the opportunity to discuss their feelings in a supportive group (Lillyman *et al.* 2011:183). In a study by Liu *et al.* (2011:856), experiential learning was also found to increase students’ motivation to learn.

Some students who participated in this study expressed existential awareness as they realised the different values people assign to dying and clarified their own values related to death and dying. They reflected on dying as part of life, similar to nursing students exposed to end-of-life curriculum content in an USA study (Dobbins 2011:164) who ‘demonstrated an increased acceptance of death as an inevitable reality of life’. Students valued discussions on the meaning of suffering and death which is sometimes covered in modules such as philosophy and ethics (Mutto *et al.* 2012:95). Nurses need to be aware of how their personal beliefs and cultural considerations may affect their responses towards a dying patient (Peters *et al.* 2013:20) and their willingness to learn about end-of-life-care (Todaro-Franceschi 2011:318).

The results suggested nursing students’ perceived clinical competency. The use of cinemeducation as a strategy of facilitating learning yielded similar results as students experienced a decrease in fear of death and increased comfort to care for dying patients (Dobbins 2011:164). Film can serve as learning support to enhance reflection and the understanding of experiences of health and suffering in the caring sciences. A film that has the potential to transform theory into a life world experience transcends the learning situation (Hörberg & Ozolins 2012:1).

### Practical implications

The results indicate that students who actively participate in learning opportunities experience sensory and emotional awareness. Nurse educators need to plan learning opportunities that will facilitate learning about end-of-life- care to raise students’ emotional awareness and provide opportunities for students to clarify personal values about death and dying. The learning opportunities need to be followed by open discussions focusing on emotional intelligence: ways to express and manage the complicated emotions nurses may experience during the provision of end-of-life care once in practice.

## Limitations of the study

In this study only qualitative data were collected. The data provided in-depth insight into the students’ learning experiences, but quantitative data, for example student questionnaires to rate the facilitation of learning, could have provided the researcher with an opportunity to identify areas of progress and areas to improve on.

The findings did not illuminate real-life experiences. It would have been helpful to assess how the experiential learning opportunities affected students’ real-life encounters with death and dying in clinical practice.

## Recommendations

A variety of innovative educational methods can be utilised to facilitate experiential learning to teach end-of-life care. Nurse educators need to use creative experiential learning opportunities to ensure students participate and use their senses during learning end-of-life care. The learning opportunities should also be designed to facilitate emotional awareness and invite students to clarify their values in relation to death and dying.

It is essential for nurse educators to obtain feedback (both qualitative and quantitative) on students’ learning experiences. These findings need to be published so that other educators can benefit from the process. Research in different areas of nursing education and the use of different educational methods will enhance the quality of nursing education. Action research is proposed as an appropriate research design to accomplish exactly this.

## Conclusion

Experiential learning opportunities opened doors for students and the nurse educator in question responsible for facilitating learning. Participants’ creative presentations facilitated their own and other participants’ learning. The reflections illustrated participants’ constructivist learning experiences that enhanced the construction of meaning. Participants experienced heightened emotional awareness as they reflected on their own encounters with death and dying. Participants were challenged to clarify their values, but realised their obligation to respect others’ values. They perceived themselves as more competent to care for dying patients and bereaved families with compassion and empathy.
